# Association between greenspace exposure before, during, and after pregnancy and autism spectrum disorder in offspring

**DOI:** 10.1038/s41370-025-00834-7

**Published:** 2026-02-27

**Authors:** Bethsaida Cardona, Hayon Michelle Choi, Kristen Lyall, Jaime E. Hart, Peter James, Marc G. Weisskopf

**Affiliations:** 1 Department of Epidemiology, Harvard TH Chan School of Public Health, Boston, MA USA; 2 Department of Environmental Heath, Harvard TH Chan School of Public Health, Boston, MA USA; 3 AJ Drexel Autism Institute, Drexel University, Philadelphia, PA USA; 4 Channing Division of Network Medicine, Department of Medicine, Brigham and Women’s Hospital and Harvard Medical School, Boston, MA USA

**Keywords:** Greenspace, Epidemiology, Maternal and fetal exposure/health, Neurodevelopment, Vulnerable Populations

## Abstract

**Background:**

Greenspace exposure in the period surrounding pregnancy may influence autism spectrum disorder (ASD) risk in offspring by reducing risk factors or mitigating effects through various pathways. Current research is limited but suggestive.

**Objective:**

We explored the association between greenspace exposure during pregnancy and ASD risk, assessing potential periods of susceptibility: 3 months preconception; first, second, and third trimester; and 3 months post-birth.

**Methods:**

We conducted a nested case-control study within the Nurses’ Health Study II (NHSII), a US prospective cohort followed up biennially. Cases were children of NHSII participants who were maternally reported to have ASD (*n* = 245). Controls were randomly selected from the full cohort and frequency matched to cases by birth year (*n* = 1526). Greenspace exposure was assessed using the satellite-based normalized difference vegetation index (NDVI) measured within 270 m and 1230 m radial buffers of the residential address of participants. Temporally matched, time-linked NDVI was used to calculate greenspace exposure for each potential period of susceptibility. Multivariable adjusted logistic regression models were applied to obtain effect estimates.

**Results:**

Greenspace exposure during pregnancy was inversely associated with ASD risk when NDVI was measured within a 270 m radial buffer of the residential address. Specifically, an interquartile range (0.144) increase in NDVI during the first trimester decreased the odds of ASD by 25% (odds ratio=0.75, 95% confidence interval: 0.56, 0.99) in a model adjusting for other time periods of exposure. There was no other 3-month exposure period significantly associated with ASD. Adjusting for PM_2.5_ did not change results. The analysis of NDVI measured within a 1230 m radial buffer showed weaker and inconsistent associations.

**Significance:**

This study found that greenspace exposure during pregnancy was inversely associated with ASD, with the first trimester being a critical exposure period. Implications for urban and city design provide compelling reasons to increase research in this field.

**Impact statement:**

Ours is the first study to report an inverse association between greenspace exposure during pregnancy and autism spectrum disorder risk in offspring that was specific to the first trimester. An interquartile range increase in satellite-based normalized difference vegetation index exposure (0.114) within a 270 m radial buffer of the residential address decreased the odds of ASD by 25% (odds ratio=0.75, 95% confidence interval: 0.56, 0.99). Future research is warranted to confirm these findings in other populations and explore the pathways by which greenspace may mitigate risk. Implications for urban and city design provide compelling reasons to increase research in this field.

## Introduction

Autism spectrum disorder (ASD) is a neurological and developmental disorder characterized by challenges in social interaction and communication, and restrictive or repetitive behaviors [1]. These behaviors typically appear in the first three years of life and can affect individuals throughout the life course. In 2020, ASD was estimated to affect 1 in 36 children in the United States (US) aged 8 years [2]. Although ASD is a well-studied and characterized disorder, the etiology remains inconclusive. Current evidence points to a strong genetic component with sizeable heritability estimates [3–5] and a growing list of identified risk genes and genetic variants [6, 7]. However, there is increasing evidence of epigenetic and environmental factors that may increase ASD independently or in conjunction with genetic factors [6, 8, 9].

Access to greenspace, such as trees or other vegetation, has limited research in relation to ASD, but may possibly reduce ASD risk factors or mitigate effects through several pathways. For example, some research shows that maternal stress is associated with ASD [6]. It has been previously reported that greenspace can improve health through physiological restoration such as attention restoration and physiological stress recovery [10, 11]. Previous research has also linked ASD with maternal obesity and diabetes [6], and greenspace may reduce this risk by promoting physical activity [10, 11]. Greenspace may also buffer exposure to air pollution, which has been associated with ASD [11–14].

Few studies have explored the association between greenspace exposure and ASD risk. A 2017 ecological study based in California found associations between various greenspace measures (percentages of forest and grassland, average tree canopy, and average near-road tree canopy) and lower autism prevalence in school districts [15]. A 2022 birth cohort study based in Vancouver, Canada showed an inverse association between greenspace and ASD, finding higher prenatal (i.e. during gestation) greenspace exposure (as measured with the satellite-based normalized difference vegetation index [NDVI]) associated with small reductions in risk of ASD development in children [16]. A 2024 matched case-control study based in Shanghai, China similarly reported an inverse association between greenspace exposure during the year before birth and the first three years after birth with ASD, although the inverse association during the first three years of life vanished after adjusting for prenatal greenspace exposure [17]. These findings support that prenatal greenspace exposure, in particular, could be inversely associated with ASD. However, a 2024 Canadian matched case-control study based in Ontario, Canada, found that while proximity to parks at birth was inversely associated with ASD, neither NDVI, eye-level street greenspace exposure using Google Street View images, nor tree canopy were associated with ASD [18]. Although both Canadian studies used large administrative datasets that are expected to have high statistical power, there could have been possible misclassification of greenspace exposure during pregnancy from use of conception year annual average NDVI [16] or birth year growing season maximum NDVI [18].

Due to limited research but a suggestive inverse association, we therefore aimed to assess the association between greenspace exposure during pregnancy and ASD, and improve on the accuracy of the exposure measurement used in previous studies by using temporally matched and time-varying NDVI data linked to participant residential address. Given that core features of ASD typically emerge in the first two years of life indicating prenatal or early postnatal origins [19–21] and the multistage development of ASD spanning the entire prenatal period [22], we aimed to assess potential periods of susceptibility, considering 3 months preconception; first, second, or third trimester; and 3 months post-birth.

## Methods

### Population/outcome measurement

We used data from a nested case-control study within the Nurses’ Health Study II (NHSII), a prospective US cohort of 116,429 female nurses aged 25–43 years when recruited in 1989, followed up by biennially mailed questionnaires. The study protocol was approved by the Institutional Review Boards of the Brigham and Women’s Hospital and the Harvard T.H. Chan School of Public Health in accordance with all relevant guidelines and regulations. The return of the completed questionnaires indicated informed consent to participate in the study. Full details of the case-control study have been described elsewhere [13, 23, 24] and briefly here.

In the 2005 biennial questionnaire, nurses reported if they had a child with autism, Asperger’s syndrome, or other autism spectrum disorder, which was combined into ASD. A case-control follow-up study was then initiated in 2007 (pilot study), followed shortly by a larger-scale study in 2009 (full study). The nested case-control study followed up on children whose mothers (the nurses) reported them as having an ASD diagnosis in the 2005 questionnaire (the cases). Using the 2005 questionnaire, controls were randomly selected from the children whose mothers did not report them as having ASD and frequency matched at a 1:4 ratio by the years in which case mothers reported births. As part of the case-control follow-up study, a supplementary questionnaire was sent to the mothers of identified cases (*n* = 756) and controls (*n* = 3000) that asked about the child’s sex, birth date, adoption status, and ASD diagnosis by a medical professional. The nurses were specifically asked if their child had autistic disorder, Asperger’s syndrome, pervasive developmental disorder (PDD), or pervasive developmental disorder not otherwise specified (PDD-NOS) that we combined into ASD. A total of 636 case mothers and 2747 control mothers responded to the supplementary questionnaire; 164 women, including 51 case mothers, declined to participate.

For the present study, participants were excluded if they met any of the following criteria: not willing to participate in the study, were adopted, had a genetic syndrome associated with ASD (fragile X syndrome, Down’s syndrome, tuberous sclerosis, trisomy 18, XXY, Jacobsen syndrome (11q deletion), or Rett’s disorder); the birth month or year were unknown, the estimated three months preconception period was prior to June 1989 (nurses’ address before this month were unknown), or address were unable to be geocoded (e.g. outside the US). Additionally, controls were excluded if birth year was outside the range of case births or if ASD was reported in the supplementary questionnaire, and cases were excluded if ASD was not reported in the supplementary questionnaire. These inclusion/exclusion criteria excluded 393 (62%) cases and 1,221 (44%) controls, leaving 245 cases and 1526 controls in the final analysis.

### Case status validation

Because ASD status was maternally reported based on questionnaires, we used the Autism Diagnostic Interview-Revised (ADI-R) [25] and Social Responsiveness Scale (SRS) [26] forms to examine the reliability of the outcome assessment. The ADI-R was administered over telephone in a subset of ASD cases from the larger case-control follow-up study [13, 23]; per recommendation, a diagnosis of autism was defined as having onset by 3 years of age and meeting the cutoff scores in all three domains of social interaction, communication and language, and restricted and repetitive behaviors [25]. Of 50 randomly selected case mothers who indicated willingness to be contacted, 43 (86%) met the ADI-R cut-off for a diagnosis of autism, 5 (10%) missed the cut-off for autism by one point on one domain, and 2 (4%) missed the cutoff in one or two domains only. The reliability of administering the ADI-R over telephone has been previously demonstrated [27]. As part of the supplementary questionnaire sent to mothers of cases and controls, mothers were also sent SRS questionnaire forms, a validated 65-item instrument designed to quantify the severity of ASD-related social traits [24]. Study participants aged 4–18 years at the time of questionnaire administration received the SRS School-Age form (SRS-2), while study participants aged 18 years or older received the SRS for Adults (SRS-A). Among our current study participants with available SRS data, 93.7% of cases (193/206) had standardized SRS T-scores of ≥ 60, indicating mild to severe autism-related traits, while 93.9% of controls (879/936) had standardized SRS T-scores of ≤ 59, indicating no clinically significant concern. Although not a clinical diagnosis tool, prior validation studies have demonstrated a high correlation between the SRS and the ADI-R [28, 29].

### Exposure

Greenspace exposure was assessed with NDVI, a satellite-based metric that indicates the quantity of vegetation on the ground. We used cloud-free composite NDVI data from the National Aeronautics and Space Administration/ US Geological Survey Landsat 5 (1984–2000) and Landsat 7 (2000–2013) satellites downloaded from Google Earth Engine at 30 m resolution. We replaced all negative NDVI values with zero, as negative values primarily represent water surfaces (which we did not want to negatively weight), such that the NDVI values ranged from 0 (indicating bare soil) to 1 (indicating dense green vegetation). The mean NDVI or standard deviation was not noticeably affected by replacing negative values with zero. NDVI was linked to the residential address of each participant, determined by the mailing address used for the biennial NHSII questionnaires. Using the addresses on file, residential-level NDVI data was downloaded once per each of four time periods in a year (January-March, April-June, July-September, and October-December) to roughly correspond with the four seasons for years 1989–2002. These years overlapped the period between 3 months preconception and 3 months post-birth for all study participants. NDVI values for each month were obtained by applying the values measured at each time period to each month in that time period. The month of conception was calculated using the birth month and year, and gestational age at birth, and then the NDVI was calculated for the entire gestational period and at each exposure period of interest (3 months preconception; first, second, and third trimester; and 3 months post-birth) by taking the average of the NDVI values for each month in the period of interest. To assess the correlation between the different exposure periods, Pearson correlations were used because NDVI was approximately normally distributed. NDVI within 270 m and 1230 m radial buffers around the geocoded addresses were measured for the analyses; a 270 m buffer was chosen to represent the immediate area around the home while a 1230 m buffer was chosen to represent the area within a 10- to 15-min walk of the home [30]. Two different buffers were chosen to offer insight about which buffer size might be most relevant, given the limited research on greenspace exposure and ASD.

### Statistical analysis

Multivariable logistic regression was used to analyze the association between greenspace and ASD risk using a continuous measure of NDVI. The association between NDVI levels and ASD risk was assessed for the entire gestational period and for each 3-month period of exposure. The different time periods of exposure were analyzed using both separate models and a mutually adjusted model incorporating all time periods to avoid co-exposure confounding. Given sex differences in ASD prevalence and diagnosis [2, 31], sex-stratified models were also conducted. However, due to lower case counts among females (*n* = 36), only effect estimates for males were reported.

All models were adjusted for potential confounders identified based on subject matter knowledge, previous research investigating the greenspace/ASD association, or previous work in this nested case control study population on air pollution [13, 16, 18]. Confounders were year of birth (continuous), maternal age at birth (continuous), paternal/partner’s education (high school or less, some college, college diploma or higher), neighborhood socioeconomic status (nSES) index (continuous), census tract population density (continuous), and geographic census region of residence (Northeast, Midwest, West, South). Of note, many individual-level variables, including maternal pregnancy complications, were not expected to confound the association between ASD and greenspace because these variables are largely unrelated to NDVI measured with satellite imagery [32]. Measures of SES were an exception because they could influence where a person lived and have been previously reported to be associated with ASD diagnosis [33, 34]. Additionally, health outcomes, such as preeclampsia and prematurity, were not adjusted as these could be mediators in the greenspace/ASD association. Paternal/partner’s education was used to account for individual-level SES. Neighborhood-level variables were based on the residential address on file closest to pregnancy. The nSES index was previously developed to differentiate neighborhood deprivation among NHS and NHSII participants [35]. US Census tract-level variables used to construct the index were obtained from the Neighborhood Change Database, which provided US Census data from 1970, 1980, 1990, 2000, and 2010 with normalized boundaries over time. The nSES index was created by *z*-standardizing and summing the following nine variables: median household income, median home value, percentage with college degree, percentage non-Hispanic White, percentage non-Hispanic Black, percentage of foreign-born residents, percentage of families receiving interest or dividends, percentage of occupied housing units, and percentage unemployed (with a higher score indicating higher nSES).

In a sensitivity analysis, models were adjusted for additional indicators of individual-level SES: race/ethnicity (non-Hispanic White, other race/ethnicity category), smoking during pregnancy (yes/no), marital status (married, never married, and other; other included divorced, separated, and widowed), and maternal grandparent education (high school or less, some college, college diploma or higher). Because previous literature has reported an association between PM_2.5_ and ASD [36–38]– including a study using the same case control cohort as us [13] – we additionally conducted separate sensitivity analyses that included PM_2.5_ as a covariate; the evidence for an association between other air pollutants and ASD is less established [36, 39]. Although we hypothesized that air pollution could mediate the greenspace/ASD association, a previous study reported no mediation effect for PM_2.5_, NO, or NO_2_ and suggested that air pollution may instead act as a confounder [16]. In our own data, we found very low associations between NDVI and PM_2.5_, suggestive of low or no mediation effect or confounding: Spearman correlations ranged from -0.07 for the entire pregnancy period to 0.11 in the 3 months preconception and first three months post birth periods. PM_2.5_ was predicted for the entire gestational period and 3-month exposure periods using a spatiotemporal generalized additive mixed model that predicted monthly outdoor concentrations at each residential address. Details of these spatiotemporal models have been previously published [13, 40]. Finally, we conducted analyses restricting the study population to the children of NHSII participants who did not change residence (non-movers) between the two questionnaires straddling pregnancy, as the exact moving date was unknown (148 cases and 894 controls). Because of the smaller sample size of confirmed non-movers, NDVI from the different exposure periods were only analyzed in separate models and not in a model that mutually adjusted for all exposure periods.

Missing data for exposure and covariates was handled using multivariate imputation by chained equations (MICE) that assumed data were missing completely at random (MCAR) or missing at random (MAR). Following guidelines by von Hippel 2020 [41], 65 datasets were imputed after accepting a 5% change in the standard error of the estimates, with 25 iterations for each imputed dataset. Separate MICE models were used for NDVI calculated with the 270 m and 1230 m buffers. All covariates used in analyses, including sensitivity analyses, were used in the MICE models, with the addition of paternal age at birth and neighborhood median income value. Rubin’s Rules were used to pool the estimates and standard errors of the imputed datasets [42]. For covariates adjusted in all models, paternal/partner’s education (2.36%), population density (3.16%), geographic region (3.16%), and nSES (3.16%) had missing data. Of covariates additionally adjusted for in the sensitivity analysis, race, marital status, maternal grandparent education, and smoking during pregnancy had missing data at 0.11%, 0.28%, 5.08%, and 24.7%, respectively. Data on PM_2.5_ was missing for the 3 months preconception period at 1.92% (*n* = 34), the first trimester at 1.64% (*n* = 29), the second trimester at 1.52% (*n* = 27), the third trimester at 2.43% (*n* = 43), the first three months of life at 0.9% (*n* = 16), and the entire gestational period at 1.64% (*n* = 29). There was also missing NDVI data (for both the 270 m and 1230 m buffers) for the 3 months preconception period at 8.75% (*n* = 155), the first trimester at 8.47% (*n* = 150), the second trimester at 8.02% (*n* = 142), the third trimester at 7.8% (*n* = 138), the first three months of life at 6.09% (*n* = 108), and the entire gestational period at 10.12% (*n* = 179). A comparison of the characteristics of participants missing any NDVI data (*n* = 213: 184 cases and 29 controls) versus not missing NDVI data (*n* = 1,566: 1340 controls and 216 cases) was reported in Supplementary Table 1, showing significant differences in missingness dependent on birth year, geographic region of residence, population density, nSES, marital status, maternal age at birth, and mover status. Some of these differences are likely due to varying satellite coverage by region.

We used SAS software Version 9 (SAS Institute, Cary, North Carolina, USA) for data extraction, and R version 4.2.0 (R Core Team, 2022) for statistical analyses.

## Results

### Participant characteristics

Our nested-case control study included 245 ASD cases and 1526 controls. Consistent with previous studies [31], the majority of ASD cases were male (85% males vs. 15% females). When compared with ASD controls, there was a higher prevalence of prematurity (18% cases vs. 15% controls), maternal gestational diabetes (6.9% cases vs. 5.7% controls), maternal preeclampsia (5.7% cases vs. 2.8% controls), and maternal smoking during pregnancy (9% cases vs. 3.3% controls) among ASD cases (Supplementary Table 2). As expected from having matched by year, the median year of birth was 1993 with a range between 1990 and 2002 for both cases and controls. Race/ethnicity, maternal age at birth, paternal age at birth, and birthweight were also comparable across groups. Compared with controls, however, cases were more likely to be from the Northeast (40% cases vs. 33% controls) and less likely to be from the South (11% cases vs. 16% controls). There was a higher mean nSES index value among the cases, indicating lower neighborhood deprivation, and a higher mean census population density (Supplementary Table 2).

### Greenspace exposure

The NDVI within 270 m of the geocoded address ranged from 0.014 to 0.60 for the entire gestational period, with an IQR of 0.144. Individual trimesters had more variability with IQRs of 0.21, 0.22, and 0.21 for the first, second, and third trimesters, respectively. The IQRs were similar for the 1230 m buffer. The Pearson correlations for the NDVI across different exposure periods were low to moderate, indicating variability within periods (Supplementary Fig. 1). However, the periods for 3 months preconception and first 3 months post-birth had the highest correlations with a coefficient of 0.77 (Supplementary Fig. 1), likely due to the annual cyclical nature of greenspace. The correlations were similar for both buffer sizes using complete case and following imputation for missing data (Supplementary Fig. 1). When comparing the correlation of the NDVI measured using the 270 m and the NDVI measured using the 1230 m buffer, the Pearson correlation was 0.85 for the entire pregnancy period and 0.9 for all 3-month periods.

Compared to participants with a lower prenatal NDVI exposure, those with a higher exposure tended to be identified as non-Hispanic White, lived in more densely populated neighborhoods, and lived in neighborhoods with higher nSES (Table 1). Trends also showed increased prenatal greenspace exposure in the South and decreased prenatal greenspace exposure in the West.

**Table 1:** Participant baseline characteristics by quartiles of residential-linked normalized difference vegetation index (NDVI) exposure during the full pregnancy.

Characteristics	1st Quartile, *N* = 398^a^	2nd Quartile, *N* = 398^a^	3rd Quartile, *N* = 398^a^	4th Quartile, *N* = 398^a^
	NDVI: 0.015–0.26	NDVI: 0.26-0.33	NDVI: 0.33-0.41	NDVI: 0.41-0.60
**Sex**				
Female	161 (40%)	176 (44%)	182 (46%)	172 (43%)
Male	237 (60%)	222 (56%)	216 (54%)	226 (57%)
**Year of Birth**				
Mean (SD)	1993.6 (2.8)	1993.8 (2.8)	1994.1 (2.8)	1994.7 (3.2)
**Race/Ethnicity**				
Other race/ethnicity category	40 (10%)	31 (7.8%)	19 (4.8%)	13 (3.3%)
Non-Hispanic White	357 (90%)	367 (92%)	378 (95%)	385 (97%)
(Missing)	1 (0.3%)	0 (0%)	1 (0.3%)	0 (0%)
**Maternal Age at Birth**				
Mean (SD)	33.7 (3.9)	33.9 (3.7)	33.6 (3.7)	34.4 (3.6)
**Paternal Age at Birth**				
Mean (SD)	36.3 (5.3)	36.6 (4.9)	36.1 (4.9)	37.2 (4.8)
(Missing)	47 (11.8%)	48 (12.1%)	38 (9.5%)	46 (11.6%)
**Maternal Marital Status**				
Married	284 (71%)	303 (76%)	289 (73%)	308 (77%)
Never Married	91 (23%)	69 (17%)	92 (23%)	61 (15%)
Other	23 (5.8%)	23 (5.8%)	16 (4.0%)	28 (7.0%)
(Missing)	0 (0%)	3 (0.8%)	1 (0.3%)	1 (0.3%)
**Paternal/Partner’s Education**				
High School or Less	54 (14%)	63 (16%)	52 (13%)	52 (13%)
1-3 Years College	61 (15%)	57 (14%)	53 (13%)	54 (14%)
4 Years College or More	261 (66%)	258 (65%)	278 (70%)	272 (68%)
Not Applicable	16 (4.0%)	5 (1.3%)	7 (1.8%)	11 (2.8%)
(Missing)	6 (1.5%)	15 (3.8%)	8 (2.0%)	9 (2.3%)
**Maternal Grandparent Education**				
High School or Less	171 (43%)	174 (44%)	189 (47%)	183 (46%)
Some College	93 (23%)	99 (25%)	77 (19%)	84 (21%)
College Diploma or Higher	114 (29%)	106 (27%)	116 (29%)	111 (28%)
(Missing)	20 (5.0%)	19 (4.8%)	16 (4.0%)	20 (5.0%)
**Region**				
Northeast	148 (37%)	145 (36%)	145 (36%)	160 (40%)
Midwest	80 (20%)	130 (33%)	149 (37%)	135 (34%)
South	23 (5.8%)	60 (15%)	73 (18%)	86 (22%)
West	140 (35%)	53 (13%)	28 (7.0%)	13 (3.3%)
(Missing)	7 (1.8%)	10 (2.5%)	3 (0.8%)	4 (1.0%)
**Population Density (persons per sq. km)**				
Mean (SD)	3917 (7231)	1425 (1461)	924 (889)	549 (777)
(Missing)	7 (1.8%)	10 (2.5%)	3 (0.8%)	4 (1.0%)
**nSES**				
Mean (SD)	1.1 (4.2)	0.7 (3.5)	0.5 (3.5)	0.3 (3.7)
(Missing)	7 (1.8%)	10 (2.5%)	3 (0.8%)	4 (1.0%)
**Birth Weight (pounds)**				
Mean (SD)	7.12 (1.38)	7.17 (1.31)	7.27 (1.30)	7.22 (1.43)
(Missing)	25 (6.3%)	49 (12.3%)	33 (8.3%)	35 (8.8%)
**Premature Birth**				
No	270 (68%)	301 (76%)	301 (76%)	277 (70%)
Yes	70 (18%)	51 (13%)	53 (13%)	67 (17%)
(Missing)	58 (15%)	46 (12%)	44 (11%)	54 (14%)
**Gestational Diabetes**				
No	310 (78%)	319 (80%)	317 (80%)	322 (81%)
Yes	24 (6.0%)	26 (6.5%)	27 (6.8%)	19 (4.8%)
(Missing)	64 (16%)	53 (13%)	54 (14%)	57 (14%)
**Maternal Preeclampsia**				
No	319 (80%)	337 (85%)	331 (83%)	328 (82%)
Yes	15 (3.8%)	8 (2.0%)	13 (3.3%)	13 (3.3%)
(Missing)	64 (16%)	53 (13%)	54 (14%)	57 (14%)
**Smoking During Pregnancy**				
No	272 (68%)	282 (71%)	296 (74%)	280 (70%)
Yes	23 (5.8%)	16 (4.0%)	11 (2.8%)	18 (4.5%)
(Missing)	103 (26%)	100 (25%)	91 (23%)	100 (25%)
**Moved**				
No	197 (49%)	246 (62%)	229 (58%)	260 (65%)
Yes	190 (48%)	142 (36%)	157 (39%)	134 (34%)
(Missing)	11 (2.8%)	10 (2.5%)	12 (3.0%)	4 (1.0%)

*SD* standard deviation, *Sq. km* square kilometer, *nSES* neighborhood socioeconomic status.

^a^Mean (SD) for continuous variables and frequency (%) for categorical variables.

179 participants had missing data on exposure and are not represented here.

### Association between NDVI and Odds of ASD

Greenspace exposure during pregnancy measured using the NDVI within a 270 m radial buffer of the residential address showed an inverse association with ASD. In adjusted models, we observed that an IQR (0.144) increase in NDVI during all pregnancy was associated with a 17% lower odds of ASD diagnosis (odds ratio [OR] = 0.83, 95% confidence interval [CI]: 0.65, 1.05) (Table 2). When restricted to males, the association was comparable (OR = 0.89, 95% CI: 0.67, 1.20). Sensitivity analyses that adjusted for additional covariates had similar effect estimates (Supplementary Table 3), while analyses that were restricted to non-movers had stronger effect estimates, albeit with wider confidence intervals (Supplementary Table 4), likely due to the more limited statistical power of the smaller sample size. Including PM_2.5_ as a covariate did not change the effect estimates for analysis combining both sexes or restricting to males (Supplementary Table 5).

**Table 2:** Odds ratios (OR) and 95% confidence intervals (CI) for the association between autism spectrum disorder and an interquartile range increase (0.144) in the residentially-linked normalized difference vegetation index.

	Whole study population^a^ OR (95% CI)		Restricted to males^b^ OR (95% CI)	
	Separate Exposure Models	Mutually Adjusted Exposure Model	Separate Exposure Models	Mutually Adjusted Exposure Model
270 m radial buffer				
Full Pregnancy	0.83 (0.65, 1.05)	-	0.89 (0.67, 1.20)	-
3 Months Preconception	0.87 (0.72, 1.05)	0.93 (0.70, 1.22)	0.86 (0.69, 1.07)	0.85 (0.62, 1.16)
1^st^ Trimester	0.77 (0.63, 0.93)	0.75 (0.56, 0.99)	0.79 (0.63, 1.00)	0.76 (0.55, 1.06)
2^nd^ Trimester	0.92 (0.76, 1.11)	1.02 (0.79, 1.34)	0.98 (0.78, 1.24)	1.09 (0.79, 1.50)
3^rd^ Trimester	1.04 (0.85, 1.26)	1.06 (0.80, 1.40)	1.10 (0.88, 1.39)	1.11 (0.80, 1.55)
3 Months Post-Birth	1.00 (0.82, 1.21)	1.12 (0.85, 1.49)	1.03 (0.82, 1.29)	1.15 (0.84, 1.59)
1230 m buffer				
Full Pregnancy	0.96 (0.76, 1.23)	-	0.98 (0.73, 1.32)	-
3 Months Preconception	0.87 (0.72, 1.06)	0.84 (0.64, 1.10)	0.81 (0.64, 1.01)	0.74 (0.54, 1.00)
1^st^ Trimester	0.87 (0.72, 1.06)	0.90 (0.68, 1.18)	0.90 (0.71, 1.13)	1.00 (0.74, 1.36)
2^nd^ Trimester	1.01 (0.83, 1.22)	1.00 (0.76, 1.31)	1.02 (0.81, 1.29)	0.97 (0.71, 1.33)
3^rd^ Trimester	1.11 (0.92, 1.35)	1.18 (0.90, 1.56)	1.11 (0.88, 1.41)	1.26 (0.90, 1.76)
3 Months Post-Birth	1.00 (0.83, 1.22)	1.06 (0.81, 1.38)	0.97 (0.77, 1.21)	1.03 (0.76, 1.39)

Effect estimates shown for the full pregnancy and at potential periods of susceptibility, including three months preconception, 1^st^ trimester, 2^nd^ trimester, 3^rd^ trimester, and 3 months post-birth. The associations for the periods of susceptibility were analyzed in separate exposure models and in mutually adjusted exposure models. Results for the entire study population and restricted to males shown.

^a^*N* = 1771; 245 cases and 1526 controls.

^b^*N* = 1000; 209 cases and 791 controls.

Logistic regression models were adjusted for: year of birth (continuous); month of birth (categorical); maternal age at birth (continuous); paternal/partner’s education (categorical); neighborhood social economic status index (continuous); population density (continuous); geographic region (categorical).

For NDVI within a 270 m radial buffer measured at different 3-month exposure periods, higher greenspace exposure during the first trimester was inversely associated with ASD, with a 23% decrease in the odds of ASD associated with an NDVI IQR increase (OR = 0.77, 95% CI: 0.63, 0.93); effects during other time periods were weaker. In models mutually adjusting for the different exposure periods (Table 2), the inverse association with first trimester NDVI persisted (OR = 0.75, 95% CI: 0.56, 0.99) while other periods were all generally null. Effects were also similar in models restricted to males (Table 2) and in sensitivity analyses adjusting for additional confounders (Supplementary Table 3), including PM_2.5_ (Supplementary Table 4). The association in the first trimester (in mutually adjusted models) was slightly attenuated when restricted to non-movers, with an OR of 0.80 (95% CI: 0.61, 1.04) overall and 0.84 (95% CI: 0.62, 1.14) when restricted to males (Supplementary Table 4). No other 3-month exposure period was statistically significantly associated with ASD diagnosis, although among non-movers greenspace exposure during the second trimester was inversely associated with ASD when both sexes were combined (OR = 0.79, 95% CI: 0.60, 1.04) and when restricted to males (OR = 0.77, 95% CI: 0.56, 1.07), but neither reached statistical significance (Supplementary Table 4).

Using NDVI within a 1230 m radial buffer of the residential address, greenspace exposure during the entire pregnancy period was not associated with ASD (OR = 0.96, 95% CI 0.76, 1.23) (Table 2), with similar results in analyses mutually adjusting for exposure periods, restricting to males, and sensitivity analyses (Table 2, Supplementary Tables 3-5). When considering individual 3-month periods, the 3 month preconception period appeared to be inversely associated for males, with an OR of 0.81 (95% CI: 0.64, 1.01) in models assessing the exposure periods separately and 0.74 (95% CI: 0.54, 1.00) in the mutually adjusted model (Table 2); the sensitivity analyses adjusted for additional covariates showed similar results (Supplementary Tables 3 and 5), but not analyses restricted to non-movers (3-month pre-conception OR = 1.00, 95% CI: 0.73, 1.35; Supplementary Table 4).

## Discussion

In this nested case-control study assessing the association between greenspace exposure before, during, and after pregnancy and ASD diagnosis in offspring, greenspace exposure during pregnancy was inversely associated with ASD when NDVI was measured within a 270 m radial buffer of the residential address. Specifically, greenspace exposure during the first trimester was associated with a 25% decrease in the odds of ASD in a model adjusting for other time periods of exposure. No other 3-month exposure period (i.e. 3 months preconception, second trimester, third trimester, or 3 months post-birth) was associated with ASD diagnosis. In analyses restricted to males, effect estimates were somewhat attenuated, with exposure during the full pregnancy period associated with a 11% decrease in odds of ASD, and exposure during the first trimester associated with a 24% decrease in odds. Analysis of NDVI measured within a 1230 m radial buffer showed weaker and inconsistent associations. Adjusting for PM_2.5_ did not change effect estimates.

Our results are consistent with Pagalan et al. [16] and Chen et al. [17], who reported that greenspace exposure during pregnancy as measured with NDVI was inversely associated with ASD in a Canadian population-based birth cohort and a Chinese matched case-control population, respectively; however, our effect estimates were considerably larger in magnitude. With an IQR unit of 0.144 used in the current study, Pagalan et al. reported a decrease in the odds of ASD of approximately 5% (OR = 0.95, 95% CI: 0.88, 1.02) within a 250 m buffer of the residential postal code and approximately 7% (OR = 0.93, 95% CI: 0.87, 1.00) within a 100 m buffer [16]; Chen et al. reported a decrease in the odds of ASD of approximately 8% (OR = 0.92, 95% CI: 0.90, 0.95) within a 500 m buffer of the residential address [17]. When comparing the effect estimates of our study during the entire pregnancy period using the 270 m buffer with Pagalan et al. using the 250 m buffer and Chen et al. using the 500 m buffer, the difference in the magnitude of the estimates is 3.4-fold and 2.1-fold. The stronger effect size could partially be explained by random sampling variability given our smaller sample size. Alternatively, the present study’s use of temporally matched and time-varying NDVI measurements linked to the residential address probably improved the accuracy of the exposure measurement over Pagalan et al. [16] and Chen et al. [17], who respectively used the conception year annual average NDVI linked to the residential postal code and the annual average NDVI during the year prior to birth. Increased non-differential exposure misclassification could be consistent with the effect estimates these prior studies reported that were closer to the null of no association. Similarly, for Lavigne et al. [18], the use of the birth year growing season maximum NDVI value to represent greenspace exposure during pregnancy might explain why they found no association between greenspace exposure using NDVI and ASD.

A novel contribution of the current study was the assessment of different 3-month exposure periods to identify critical periods of greenspace exposure, finding that increased greenspace exposure during the first trimester was inversely associated with ASD risk. The first trimester coincides with rapid neurulation, neuronal proliferation, and some neuronal migration [43, 44]. That we identified the first trimester as a period of susceptibility in models regarding the exposure periods separately and in mutually adjusted models reinforced our results; observing different effect estimates when including other exposure periods may indicate residual confounding by time-invariant confounders related to the different exposure periods [45–47]. Conversely, the effect estimates for the third trimester and 3 months post-birth were higher in magnitude in the mutually adjusted models, raising the possibility of co-exposure amplification bias, which occurs when there is residual confounding of correlated exposure variables in regression models [48]. However, the likelihood of confounding of a residentially-based NDVI measure that is specific to those time periods seems less likely, in which case co-exposure amplification bias would not occur. Nonetheless, cautious interpretation is suggested for the effect estimates corresponding to the third trimester and 3 months post-birth exposure periods.

Notably, the current results showing the first trimester to be an important period of susceptibility for greenspace exposure in influencing ASD risk is in contrast to studies on air pollution that have reported the third trimester and early childhood to be critical periods of susceptibility [49], including a study using the same nested-case control population as the present study [13]. This contrast may indicate different biological mechanisms whereby pathways facilitated by greenspace, such as increased physical activity, may be more important in the earlier periods of pregnancy. Although we theorized that air pollution may mediate the greenspace/ASD association, we did not find an association between PM_2.5_ and greenspace in our study and adjusting for PM_2.5_ did not change effect estimates, suggestive of a weaker mediation or confounding effect. Pagalan et al. [16] and Chen et al. [17] also did not find a statistically significant mediating effect by air pollutants, including by PM_2.5_, PM_10_, PM_1_, NO, or NO_2_, To provide further insight into the relevance of the first trimester, studies are needed to explore sensitive periods of exposure for the hypothesized protective benefits of greenspace, such as increased physical activity and decreased maternal stress.

While we also reported NDVI within a larger 1230 m radial buffer, mostly null results suggested that this larger buffer size for measuring NDVI could be inadequate for assessing the greenspace/ASD association. Previous studies have demonstrated that the choice of buffer size influences study results [30, 50], including in the greenspace-ASD literature: Pagalan et al. [16] found that prenatal greenspace exposure measured using NDVI within a 100 m buffer was significantly associated with ASD, but 250 m and 500 m buffers had slightly more attenuated results that did not reach statistical significance [16]. Differences in results may be due to larger radial buffers capturing natural features of the environment, such as highways and mountains, that are inaccessible to study participants and mask how individuals interact with greenspace. Nevertheless, further consideration is warranted for our findings of an inverse association during the preconception period in analyses restricted to males when using NDVI measured within a 1230 m radial buffer, but not within a 270 m buffer.

### Strengths and limitations

This study has several significant strengths. To our knowledge, this is the first study to identify the first trimester of pregnancy as a potential period of susceptibility using a nationwide US study population. Our study extends the applicability of previous study findings that were based in Canada [16, 18], California [15], or China [17]. The present study’s use of temporally matched and time-varying NDVI data linked to the residential address may have also improved the accuracy of the exposure measurement compared to previous studies [16–18]. Additionally, temporally changing NDVI data enabled exploring different time periods of exposure surrounding the pregnancy period, which had not been previously done. These findings open additional venues of research that could confirm study results or explore potential mechanisms. We also reported on two different radial buffer sizes that could aid future research in identifying buffers. The various sensitivity analyses conducted indicated robust study results.

The current study also has limitations. The smaller sample size might lack power, potentially leading to some of our null observations and to effect estimates that were larger than in previous studies [16, 17]. The generalizability of our study findings could also be limited given differences in diagnostic criteria, autism awareness, and environmental greenspace contexts. However, to the extent that more cases were missed in the years of our data compared to now when rates have gotten higher, this would likely have biased any effect towards the null rather than create a spurious association. While many important confounders were considered, the possibility of residual confounding cannot be ruled out, including from individual-level SES and environmental factors. There may also be some inaccuracy or imprecision in the exposure measurement. For example, 38% of the study population moved residence in between questionnaire periods, increasing the likelihood of exposure misclassification if data from the inappropriate address was used. However, we found consistent results when we restricted to non-movers increasing the validity of our study findings. In addition, exposure misclassification is expected to bias results towards the null: misclassification is unlikely to be differential between cases and controls as the same methodology for measuring NDVI was used for all participants without knowledge of case status. Because we used maternally reported ASD status, there may also be some outcome misclassification. However, case status was validated among a subset of cases showing high accuracy [23], there was strong alignment between case status and autism-related traits measured with SRS scores, and the mothers’ nursing degrees may enable them to provide more accurate self-reported information [51]. In using NDVI as a measure of greenspace, we were not able to capture the quality of greenspace or explore whether certain features of greenspace, like parks or gardens, were more protective than others.

Future studies could significantly benefit from using different metrics for greenspace that are more specific (e.g. percent trees or percent grass [52–54]) and could better inform policy recommendations when considering urban and city design. It is also possible that certain greenspaces have higher levels of pesticides, with some studies linking pesticide exposure to ASD [55, 56] that could mediate or confound the greenspace/ASD association. Additional studies should investigate the role of pesticide-containing versus pesticide-free greenspaces on ASD risk.

### Conclusion

We found that increased greenspace exposure during pregnancy was inversely associated with ASD risk, specifically during the first trimester. No other 3-month exposure period assessed was significantly associated with ASD. Future research is warranted to confirm these findings in other populations, and to explore the pathways by which greenspace may mitigate risk.

## Supplementary information

Supplementary Tables

Supplementary information

## Data Availability

Because of participant confidentiality and privacy concerns, data cannot be shared publicly and requests to access NHSII data must be submitted in writing. According to standard controlled access procedures, applications to use NHSII resources will be reviewed by our External Collaborations Committee to verify that the proposed use maintains the protection of the privacy of participants and the confidentiality of the data. Investigators wishing to use NHSII data are asked to submit a brief description of the proposed project (go to https://www.nurseshealthstudy.org/researchers (contact email: nhsaccess@channing.harvard.edu) and https://sites.sph.harvard.edu/hpfs/for-collaborators/ for details).
