# Supplementary material for: Association between greenspace exposure before, during, and after pregnancy and autism spectrum disorder in offspring
Source: J Expo Sci Environ Epidemiol. 2026 Feb 27;36(3):585–93. doi: 10.1038/s41370-025-00834-7 (PMC13143818; doi:10.1038/s41370-025-00834-7)
Supplement: Supplementary file 1 — Supplementary Tables [file 41370_2025_834_MOESM1_ESM.docx]

**Supplementary Table 1.** Participant characteristics at baseline comparing participants missing any normalized difference vegetation index (NDVI) data (either full pregnancy; first 3-months pre-conception; first, second, or third trimester; or first 3 months after birth) versus not missing any NDVI data

| **Characteristic** | **Missing data, N = 212^a^** | **Not missing data,**  **N = 1,559 ^a^** | **p-value^b^** |
| --- | --- | --- | --- |
| **Case Status** |  |  | >0.9 |
| Control | 183 (86%) | 1,343 (86%) |  |
| Case | 29 (14%) | 216 (14%) |  |
| **Maternal Grandparent Education** |  |  | 0.14 |
| High School or Less | 106 (50%) | 694 (45%) |  |
| Some College | 39 (18%) | 349 (22%) |  |
| College Diploma or Higher | 52 (25%) | 441 (28%) |  |
| (Missing) | 15 (7.1%) | 75 (4.8%) |  |
| **Sex** |  |  | >0.9 |
| Female | 93 (44%) | 678 (43%) |  |
| Male | 119 (56%) | 881 (57%) |  |
| **Paternal/Partner’s Education** |  |  | 0.8 |
| High School or Less | 26 (12%) | 215 (14%) |  |
| 1-3 Years College | 35 (17%) | 216 (14%) |  |
| 4 Years College or more | 141 (67%) | 1,051 (67%) |  |
| Not Applicable | 6 (2.8%) | 39 (2.5%) |  |
| (Missing) | 4 (1.9%) | 38 (2.4%) |  |
| **Marital Status** |  |  | <0.001 |
| Married | 189 (89%) | 1,157 (74%) |  |
| Never Married | 15 (7.1%) | 308 (20%) |  |
| Other | 8 (3.8%) | 89 (5.7%) |  |
| (Missing) | 0 (0%) | 5 (0.3%) |  |
| **Year of Birth** |  |  | <0.001 |
| Mean (SD) | 1,991.6 (2.2) | 1,994.1 (2.9) |  |
| **Maternal Age at Birth** |  |  | <0.001 |
| Mean (SD) | 32.2 (3.7) | 33.9 (3.7) |  |
| **Smoking During Pregnancy** |  |  | 0.3 |
| Yes | 5 (2.4%) | 67 (4.3%) |  |
| No | 157 (74%) | 1,104 (71%) |  |
| (Missing) | 50 (24%) | 388 (25%) |  |
| **Race/Ethnicity** |  |  | 0.7 |
| Other Race/Ethnicity Category | 11 (5.2%) | 100 (6.4%) |  |
| Non-Hispanic White | 201 (95%) | 1,457 (93%) |  |
| (Missing) | 0 (0%) | 2 (0.1%) |  |
| **Median Census House Value** |  |  | <0.001 |
| Mean (SD) | 92,513 (58,872) | 134,264 (93,912) |  |
| (Missing) | 33 (15.6%) | 23 (1.5%) |  |
| **Median Census Income** |  |  | <0.001 |
| Mean (SD) | 42,468 (14,593) | 47,491 (16,492) |  |
| (Missing) | 33 (15.6%) | 23 (1.5%) |  |
| **nSES** |  |  | <0.001 |
| Mean (SD) | -0.3 (3.1) | 0.7 (3.7) |  |
| (Missing) | 33 (15.6%) | 23 (1.5%) |  |
| **Region** |  |  | <0.001 |
| Northeast | 12 (5.7%) | 594 (38%) |  |
| Midwest | 118 (56%) | 475 (30%) |  |
| South | 36 (17%) | 237 (15%) |  |
| West | 13 (6.1%) | 230 (15%) |  |
| (Missing) | 33 (16%) | 23 (1.5%) |  |
| **Population Density** |  |  | 0.005 |
| Mean (SD) | 1,081 (2,412) | 1,719 (3,990) |  |
| (Missing) | 33 (15.6%) | 23 (1.5%) |  |
| **Moved** |  |  | <0.001 |
| No | 125 (59%) | 917 (59%) |  |
| Yes | 64 (30%) | 605 (39%) |  |
| (Missing) | 23 (11%) | 37 (2.4%) |  |

Abbreviations: IQR, interquartile range; SD, standard deviation; nSES, neighborhood socioeconomic status

^a^Mean (SD) for continuous variables and frequency (%) for categorical variables

^b^Pearson's Chi-squared test; Wilcoxon rank sum test

**Supplementary Table 2.** Participant baseline characteristics by autism spectrum disorder case status

| **Characteristics** | **Control, N=1,526^a^** | **Case, N=245^a^** |
| --- | --- | --- |
| **Sex** |  |  |
| Female | 735 (48%) | 36 (15%) |
| Male | 791 (52%) | 209 (85%) |
| **Year of Birth** |  |  |
| Mean (SD) | 1,993.8 (2.9) | 1,993.8 (2.9) |
| **Race/Ethnicity** |  |  |
| Other race/ethnicity category | 91 (6.0%) | 20 (8.2%) |
| Non-Hispanic White | 1,433 (94%) | 225 (92%) |
| (Missing) | 2 (0.1%) | 0 (0%) |
| **Maternal Age at Birth** |  |  |
| Mean (SD) | 33.7 (3.7) | 34.0 (4.0) |
| **Paternal Age at Birth** |  |  |
| Mean (SD) | 36.3 (4.9) | 36.9 (5.3) |
| (Missing) | 178 (11.7%) | 11 (4.5%) |
| **Maternal Marital Status** |  |  |
| Married | 1,162 (76%) | 184 (75%) |
| Never Married | 270 (18%) | 53 (22%) |
| Other | 89 (5.8%) | 8 (3.3%) |
| (Missing) | 5 (0.3%) | 0 (0%) |
| **Paternal/Partner’s Education** |  |  |
| High School or Less | 209 (14%) | 32 (13%) |
| 1-3 Years College | 208 (14%) | 43 (18%) |
| 4 Years College or More | 1,039 (68%) | 153 (62%) |
| Not Applicable | 35 (2.3%) | 10 (4.1%) |
| (Missing) | 35 (2.3%) | 7 (2.9%) |
| **Maternal Grandparent Education** |  |  |
| High School or Less | 685 (45%) | 115 (47%) |
| Some College | 321 (21%) | 67 (27%) |
| College Diploma or Higher | 431 (28%) | 62 (25%) |
| (Missing) | 89 (5.8%) | 1 (0.4%) |
| **Region** |  |  |
| Northeast | 508 (33%) | 98 (40%) |
| Midwest | 514 (34%) | 79 (32%) |
| South | 247 (16%) | 26 (11%) |
| West | 212 (14%) | 31 (13%) |
| (Missing) | 45 (2.9%) | 11 (4.5%) |
| **Population Density** |  |  |
| Mean (SD) | 1,556 (3,623) | 2,261 (5,080) |
| (Missing) | 45 (2.9%) | 11 (4.5%) |
| **nSES^b^** |  |  |
| Mean (SD) | 0.5 (3.7) | 0.8 (3.6) |
| (Missing) | 45 (2.9%) | 11 (4.5%) |
| **Birth Weight (pounds)** |  |  |
| Mean (SD) | 7.20 (1.34) | 7.15 (1.53) |
| (Missing) | 134 (8.9%) | 22 (9%) |
| **Premature Birth** |  |  |
| No | 1,143 (75%) | 143 (58%) |
| Yes | 225 (15%) | 43 (18%) |
| (Missing) | 158 (10%) | 59 (24%) |
| **Gestational Diabetes** |  |  |
| No | 1,223 (80%) | 188 (77%) |
| Yes | 87 (5.7%) | 17 (6.9%) |
| (Missing) | 216 (14%) | 40 (16%) |
| **Maternal Preeclampsia** |  |  |
| No | 1,267 (83%) | 191 (78%) |
| Yes | 43 (2.8%) | 14 (5.7%) |
| (Missing) | 216 (14%) | 40 (16%) |
| **Smoking During Pregnancy** |  |  |
| No | 1,103 (72%) | 158 (64%) |
| Yes | 50 (3.3%) | 22 (9.0%) |
| (Missing) | 373 (24%) | 65 (27%) |
| **Moved** |  |  |
| No | 894 (59%) | 148 (60%) |
| Yes | 575 (38%) | 94 (38%) |
| (Missing) | 57 (3.7%) | 3 (1.2%) |

Abbreviations: SD, standard deviation; nSES, neighborhood socioeconomic status

^a^Mean (SD) for continuous variables and frequency (%) for categorical variables

^b^A higher nSES indicates higher neighborhood SES

**Supplementary Figure 1.** Correlation matrices showing the Pearson correlation coefficient of the NDVI values from the different 3-month exposure periods: 3 months preconception; first, second, and third trimester; and first 3 months after birth. Pearson correlation coefficients range from -1 to 1, with values closer to -1 indicating higher negative correlation, values closer to 0 indicating no correlation, and values closer to 1 indicating higher positive correlation. Correlations are shown for the NDVI measured with the 270m radial buffer with only non-missing NDVI data (A) and with the imputed NDVI data using multivariate imputation by chained equations (MICE) (B); and for the NDVI measured using the 1,230m radial buffer with only non-missing NDVI data (C) and with the MICE-imputed NDVI data (D).

**
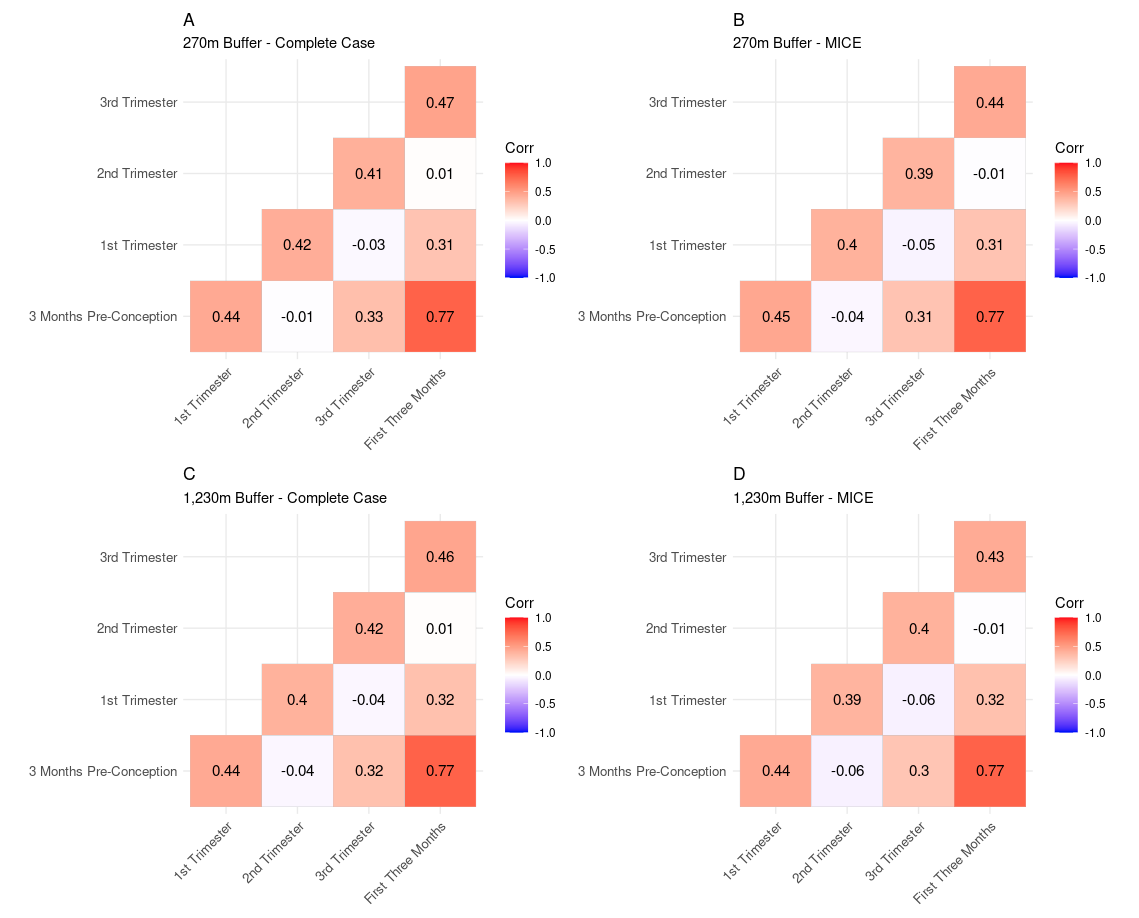
**

**Supplementary Table 3.** Sensitivity analysis adjusting for additional individual-level proxies of socioeconomic status. Odds ratios (OR) and 95% confidence intervals (CI) shown for the association between autism spectrum disorder and an interquartile range increase (0.144) in the residentially-linked normalized difference vegetation index. Effect estimates shown for the full pregnancy and at potential periods of susceptibility, including three months preconception, 1^st^ trimester, 2^nd^ trimester, 3^rd^ trimester, and 3 months post-birth. The associations for the periods of susceptibility were analyzed in separate exposure models and in mutually adjusted exposure models. Results for the entire study population and restricted to males are shown.

|  | **Whole study population**^a^  OR (95% CI) | | **Restricted to males^b^**  OR (95% CI) | |
| --- | --- | --- | --- | --- |
|  | Separate Exposure Models | Mutually Adjusted Exposure Model | Separate Exposure Models | Mutually Adjusted Exposure Model |
| 270m radial buffer | | | | |
| Full Pregnancy | 0.85 (0.66, 1.09) | - | 0.90 (0.67, 1.22) | - |
| 3 Months Preconception | 0.88 (0.73, 1.07) | 0.92 (0.69, 1.21) | 0.86 (0.68, 1.07) | 0.84 (0.61, 1.16) |
| 1^st^ Trimester | 0.79 (0.64, 0.96) | 0.77 (0.58, 1.03) | 0.80 (0.63, 1.02) | 0.78 (0.56, 1.08) |
| 2^nd^ Trimester | 0.92 (0.76, 1.13) | 1.01 (0.76, 1.33) | 0.99 (0.78, 1.26) | 1.10 (0.79, 1.52) |
| 3^rd^ Trimester | 1.05 (0.86, 1.28) | 1.07 (0.81, 1.42) | 1.09 (0.86, 1.39) | 1.09 (0.78, 1.53) |
| 3 Months Post-Birth | 1.02 (0.83, 1.25) | 1.14 (0.86, 1.51) | 1.03 (0.82, 1.30) | 1.16 (0.84, 1.61) |
| 1,230m radial buffer | | | | |
| Full Pregnancy | 0.99 (0.78, 1.27) | - | 0.99 (0.73, 1.34) | - |
| 3 Months Preconception | 0.89 (0.73, 1.08) | 0.84 (0.64, 1.10) | 0.80 (0.64, 1.01) | 0.73 (0.53, 1.00) |
| 1^st^ Trimester | 0.90 (0.74, 1.10) | 0.94 (0.71, 1.24) | 0.91 (0.72, 1.16) | 1.04 (0.76, 1.42) |
| 2^nd^ Trimester | 1.02 (0.84, 1.24) | 0.98 (0.75, 1.29) | 1.02 (0.80, 1.30) | 0.96 (0.70, 1.33) |
| 3^rd^ Trimester | 1.12 (0.92, 1.37) | 1.20 (0.90, 1.58) | 1.10 (0.86, 1.40) | 1.24 (0.88, 1.75) |
| 3 Months Post-Birth | 1.02 (0.84, 1.25) | 1.06 (0.80, 1.39) | 0.96 (0.76, 1.21) | 1.02 (0.75, 1.39) |

^a^ N=1,771; 245 cases and 1,526 controls

^b^ N=1,000; 209 cases and 791 controls

Logistic regression models were adjusted for: smoking (binary); marital status (categorical); race/ethnicity (binary); maternal grandparent education (categorical); year of birth (continuous); month of birth (categorical); maternal age at birth (continuous); paternal/partner’s education (categorical); neighborhood socioeconomic status (continuous); population density (continuous); geographic region (categorical).

**Supplementary Table 4.** Sensitivity analysis restricting to non-movers. Odds ratios (OR) and 95% confidence intervals (CI) shown for the association between autism spectrum disorder and an interquartile range increase (0.144) in the residentially-linked normalized difference vegetation index. Effect estimates shown for the full pregnancy and at potential periods of susceptibility, including three months preconception, 1^st^ trimester, 2^nd^ trimester, 3^rd^ trimester, and 3 months post-birth. The associations for the periods of susceptibility were analyzed in separate exposure models. Results for all non-movers and restricted to male non-movers are shown.

|  | **All non-movers^a^**  OR (95% CI) | **Male non-movers^b^**  OR (95% CI) |
| --- | --- | --- |
|  | Separate Exposure Models | Separate Exposure Models |
| 270m radial buffer | | |
| Full Pregnancy | 0.79 (0.56, 1.12) | 0.82 (0.54, 1.23) |
| 3 Months Preconception | 0.93 (0.72, 1.21) | 1.06 (0.79, 1.44) |
| 1^st^ Trimester | 0.80 (0.61, 1.04) | 0.84 (0.62, 1.14) |
| 2^nd^ Trimester | 0.79 (0.60, 1.04) | 0.77 (0.56, 1.07) |
| 3^rd^ Trimester | 1.14 (0.87, 1.49) | 1.15 (0.83, 1.59) |
| 3 Months Post-Birth | 1.11 (0.84, 1.46) | 1.23 (0.90, 1.69) |
| 1,230m radial buffer | | |
| Full Pregnancy | 0.98 (0.71, 1.37) | 0.92 (0.61, 1.37) |
| 3 Months Preconception | 0.96 (0.74, 1.25) | 1.00 (0.73, 1.35) |
| 1^st^ Trimester | 0.91 (0.71, 1.17) | 0.94 (0.70, 1.27) |
| 2^nd^ Trimester | 0.91 (0.70, 1.17) | 0.81 (0.60, 1.12) |
| 3^rd^ Trimester | 1.29 (0.98, 1.70) | 1.20 (0.86, 1.67) |
| 3 Months Post-Birth | 1.11 (0.85, 1.46) | 1.14 (0.83, 1.57) |

^a^ N=1,060; 148 cases and 894 controls

^b^ N=588; 129 cases and 459 controls

Logistic regression models were adjusted for: year of birth (continuous); month of birth (categorical); maternal age at birth (continuous); paternal/partner’s education (categorical); nSES (continuous); population density (continuous); geographic region (categorical).

**Supplementary Table 5.** Sensitivity analysis adjusting for fine particulate matter (PM_2.5_). Odds ratios (OR) and 95% confidence intervals (CI) for the association between autism spectrum disorder and an interquartile range increase (0.144) in the residentially-linked normalized difference vegetation index. Effect estimates shown for the full pregnancy and at potential periods of susceptibility, including three months preconception, 1^st^ trimester, 2^nd^ trimester, 3^rd^ trimester, and 3 months post-birth. The associations for the periods of susceptibility were analyzed in separate exposure models. Results for the entire study population and restricted to males are shown.

|  | **Whole study population^a^**  OR (95% CI) | **Restricted to males^b^**  OR (95% CI) |
| --- | --- | --- |
|  | Separate Exposure Models | Separate Exposure Models |
| 270m radial buffer | | |
| Full Pregnancy | 0.84 (0.66, 1.07) | 0.90 (0.67, 1.20) |
| 3 Months Preconception | 0.87 (0.71, 1.05) | 0.85 (0.68, 1.07) |
| 1^st^ Trimester | 0.76 (0.63, 0.93) | 0.78 (0.62, 0.99) |
| 2^nd^ Trimester | 0.91 (0.76, 1.11) | 0.97 (0.77, 1.23) |
| 3^rd^ Trimester | 1.05 (0.86, 1.27) | 1.12 (0.88, 1.41) |
| 3 Months Post-Birth | 1.00 (0.82, 1.22) | 1.03 (0.82, 1.29) |
| 1,230m radial buffer | | |
| Full Pregnancy | 0.97 (0.76, 1.24) | 0.98 (0.72, 1.32) |
| 3 Months Preconception | 0.87 (0.72, 1.05) | 0.80 (0.64, 1.01) |
| 1^st^ Trimester | 0.87 (0.72, 1.05) | 0.89 (0.70, 1.12) |
| 2^nd^ Trimester | 1.00 (0.83, 1.22) | 1.01 (0.80, 1.28) |
| 3^rd^ Trimester | 1.12 (0.92, 1.36) | 1.12 (0.88, 1.42) |
| 3 Months Post-Birth | 1.00 (0.82, 1.22) | 0.96 (0.77, 1.21) |

^a^ N=1,771; 245 cases and 1,526 controls

^b^ N=1,000; 209 cases and 791 controls

Logistic regression models were additionally adjusted for: year of birth (continuous); month of birth (categorical); maternal age at birth (continuous); paternal/partner’s education (categorical); nSES (continuous); population density (continuous); geographic region (categorical)
